# Occupational Diseases: From Cure to Prevention

**DOI:** 10.3390/jcm8101681

**Published:** 2019-10-14

**Authors:** Henk F. van der Molen, Monique H. W. Frings-Dresen

**Affiliations:** Amsterdam UMC, University of Amsterdam, Coronel Institute of Occupational Health, Netherlands Center for Occupational Diseases, Amsterdam Public Health Research Institute, PO Box 22660, 1100 DD Amsterdam, The Netherlands

Occupational diseases are broadly defined as diseases with a specific clinical diagnosis associated with work-related factors. The worldwide burden of work-related diseases and injuries is high, although there are large variations in and between countries [1]. A work-related disease is any health condition partly or primarily (occupational disease) due to exposure to risk factors arising from work activity [2]. In principle, work-related diseases can be prevented by means of timely control measures at worksites. This Special Issue addresses the topic of occurrence, risk factors and preventive actions across different types of occupational and work-related diseases.

Detecting early signs of work-related diseases could stimulate prevention [3]. Early signs can be defined as combinations of symptoms or signs and evidence based work-related risk factors. Mostly, work-related diseases are developed over time and early actions can prevent the status of occupational disease. Early signs of work-related diseases provide the opportunity to detect adverse outcomes due to work at an earlier stage. Information about early signs for work-related diseases can also lead to actionable risk communication with the worker or patient to reduce work-related risk factors. Early signs for occupational diseases are strengthening the urgency and preparedness of (co-)workers to take preventive actions to reduce work related risk factors. Examples of actions are stepladders for construction workers to prevent low back and shoulder disorders or adapting working schedules to prevent stress related disorders among health care workers. Clinicians are not always familiar with risk factors in the workplace related to diseases. Communications between clinicians and occupational physicians about possible risk factors related to diseases is important for sustainable work participation of workers.

Considering the factor work in clinical care is to increase knowledge of work-related risk factors in diagnoses, selective prevention and treatment in the daily care of patients. The diagnoses of occupational diseases or work-related diseases is often complex due to its multifactorial origin and dependent on work-related, personal (age, sex, genetic) and environmental factors. Work-related risk factors can worsen the prognosis and can hinder returning to work or sustaining at work. Therefore, knowledge about the work-relatedness of diseases is useful for clinicians in order to take timely and actively actions related to risk factors in the workplace; it will optimize the work participation of their patients and prevent worsening of diseases.
